# Proteomic analysis of young leaves at three developmental stages in an albino tea cultivar

**DOI:** 10.1186/1477-5956-9-44

**Published:** 2011-08-02

**Authors:** Qin Li, Jianan Huang, Shuoqian Liu, Juan Li, Xinhe Yang, Yisong Liu, Zhonghua Liu

**Affiliations:** 1 Key Laboratory of Tea Science of Ministry of Education and Hunan Provincial Key Laboratory of Crop Germplasm Innovation and Utilization, Hunan Agricultural University, Changsha, Hunan 410128, People's Republic of China; 2 National Research Center of Engineering & Technology for Utilization of Botanical Functional Ingredients, Changsha, Hunan 410128, People's Republic of China

## Abstract

**Background:**

White leaf No.1 is a typical albino tea cultivar grown in China and it has received increased attention in recent years due to the fact that white leaves containing a high level of amino acids, which are very important components affecting the quality of tea drink. According to the color of its leaves, the development of this tea cultivar is divided into three stages: the pre-albinistic stage, the albinistic stage and the regreening stage. To understand the intricate mechanism of periodic albinism, a comparative proteomic approach based on two-dimensional electrophoresis (2-DE) and mass spectrometry was adopted first time to identify proteins that changed in abundance during the three developmental periods.

**Results:**

The 2-DE results showed that the expression level of 61 protein spots varied markedly during the three developmental stages. To analyze the functions of the significantly differentially expressed protein spots, 30 spots were excised from gels and analyzed by matrix-assisted laser desorption ionization-time of flight-tandem mass spectrometry. Of these, 26 spots were successfully identified. All identified protein spots were involved in metabolism of carbon, nitrogen and sulfur, photosynthesis, protein processing, stress defense and RNA processing, indicating these physiological processes may play crucial roles in the periodic albinism. Quantitative real-time RT-PCR analysis was used to assess the transcriptional level of differentially expressed proteins. In addition, the ultrastructural studies revealed that the etioplast-chloroplast transition in the leaf cell of White leaf No. 1 was inhibited and the grana in the chloroplast was destroyed at the albinistic stage.

**Conclusions:**

In this work, the proteomic analysis revealed that some proteins may have important roles in the molecular events involved in periodic albinism of White leaf No. 1 and identificated many attractive candidates for further investigation. In addition, the ultrastructural studies revealed that the change in leaf color of White leaf No. 1 might be a consequence of suppression of the etioplast-chloroplast transition and damage to grana in the chloroplast induced by temperature. These results provide much useful information to improve our understanding of the mechanism of albinism in the albino tea cultivar.

## Background

The tea plant, *Camellia sinensis *(L.) O.Kuntze, is a perennial woody plant widely cultivated for the production of a popular non-alcoholic beverage. As one of the most famous soft drinks, tea plays a significant role in the economy of the countries where it is produced. As a major tea-producing country, China has a long history of tea cultivation and a great variety of tea germplasm of which albino cultivars, which bear white leaves containing a high level of amino acids, have received extensive attention.

White leaf No.1 (formerly Anji White) is a typical albino tea cultivar grown in China. According to the color of its leaves, development of the plant is divided into three stages: the pre-albinistic stage, albinistic stage and regreening stage. A previous study suggested that the leaf color of White leaf No.1 is sensitive to temperature. When the temperature is below 20°C at the budding stage in early spring, the leaves gradually change from light green to completely white. After about two weeks at the albinistic stage, the leaves gradually turn as green as those of common tea cultivars when the temperature rises above 22°C during the one bud and leaf stage [1]. The biosynthesis of chlorophyll was blocked and the level of chlorophyll was decreased at the albinistic stage, but it returns to normal at the regreening stage [2]. Chemical analysis of White leaf No.1 showed that in the albinistic stage the white leaves contain a high concentration of total amino acids, especially theanine, which is an amino acid found exclusively in tea [3] and is extremely beneficial for human health [4-9], but lower levels of total polyphenol and caffeine compared with green-leaved cultivars [10]. Analysis of changes in enzyme activity showed that the activity of superoxide dismutase, Rubisco large/small subunit and catalase dropped sharply at the albinistic stage, but peroxidase and proteinase activities increased at the same time. The increased activity of proteinase might be the direct reason for the accumulation of total amino acids [11,12]. A study of the pigment-protein complexes of White leaf No.1 revealed that suppression of the P700-chlorophyll *a *protein complex and light-harvesting chlorophyll *a*/*b *protein complex might be one reason for the albinism [13]. In addition, differences in gene expression between the green and albinistic leaves of White leaf No.1 were studied to elucidate the reason for the periodic albinism of White leaf No. 1 [14].

In spite of a certain amount of work on White leaf No.1, there is little proteomic information available on White leaf No.1 during shoot development. Most previous studies focused on the effects of environmental factors and physiology of White leaf No.1 on the periodic albinism. In this paper, two-dimensional electrophoresis (2-DE) was firstly used to separate proteins differentially expressed at three developmental stages of albino tea cultivars. These proteins were identified by matrix-assisted laser desorption ionization time-of-flight mass spectrometry (MALDI-TOF/TOF MS). In addition, the ultrastructure of chloroplasts at the three developmental stages was studied. The objective of these studies was to obtain an improved understanding of the albinism mechanism in an albino tea cultivar.

## Results

### Chloroplast ultrastructure at different developmental stage

**Figure 1:**
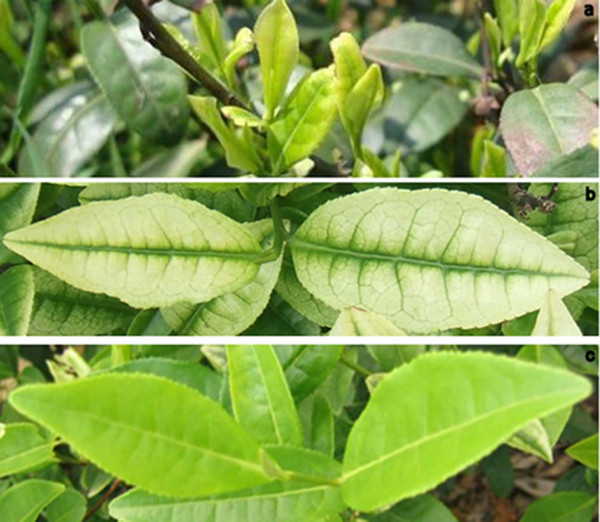
**Phenotypes of White leaf No. 1 at three developmental stages**. The pre-albinistic stage (a), albinistic stage (b) and regreening stage (c).

**Figure 2:**
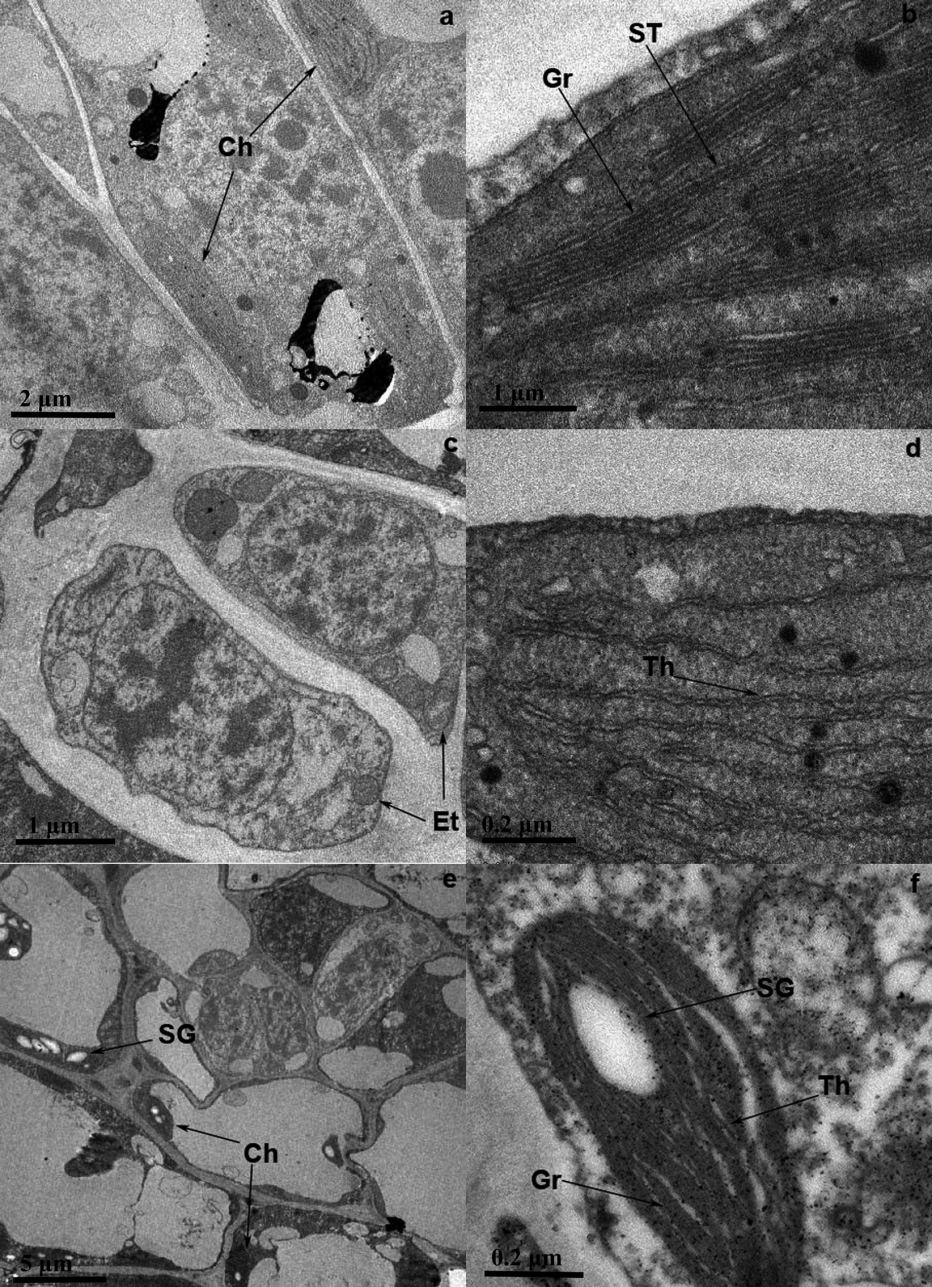
**Ultrastructur e of leaf cells at three developmental stages**. The pre-albinistic stage (a, b), albinistic stage (c, d) and regreening stage (e, f); Ch: chloroplast; Gr: grana; ST: stroma thylakoid; Et: etioplast; Th: thylakoid; SG: starch granule.

The phenotypes of White leaf No. 1 leaves at the three developmental stages are shown in Figure 1. It is well known that leaf greening is the result of chloroplast development and most of the chlorophyll is located in the grana of chloroplasts. In the pre-albinistic stage of White leaf No. 1, the chloroplasts showed the typical ultrastructure, which consisted of grana, thylakoids and stroma thylakoids (Figure 2a and 2b). Chloroplasts develop from etioplasts [15] and their development is suppressed by low temperature [16]. In the pre-albinistic stage of White leaf No. 1, etioplasts were observed though the number of mature intact chloroplasts decreased markedly (Figure 2c). The grana disappeared and only a few thylakoids remained in the chloroplast (Figure 2d). When the temperature increased in the regreening stage, the number of normal chloroplasts increased in the leaf cells and the chloroplast structure returned to normal (Figure 2e and 2f). These results indicated that the change in leaf color of White leaf No. 1 might be a consequence of suppression of the etioplast-chloroplast transition and damage to grana in the chloroplast induced by temperature.

### Change in protein abundance at different developmental stage

**Figure 3:**
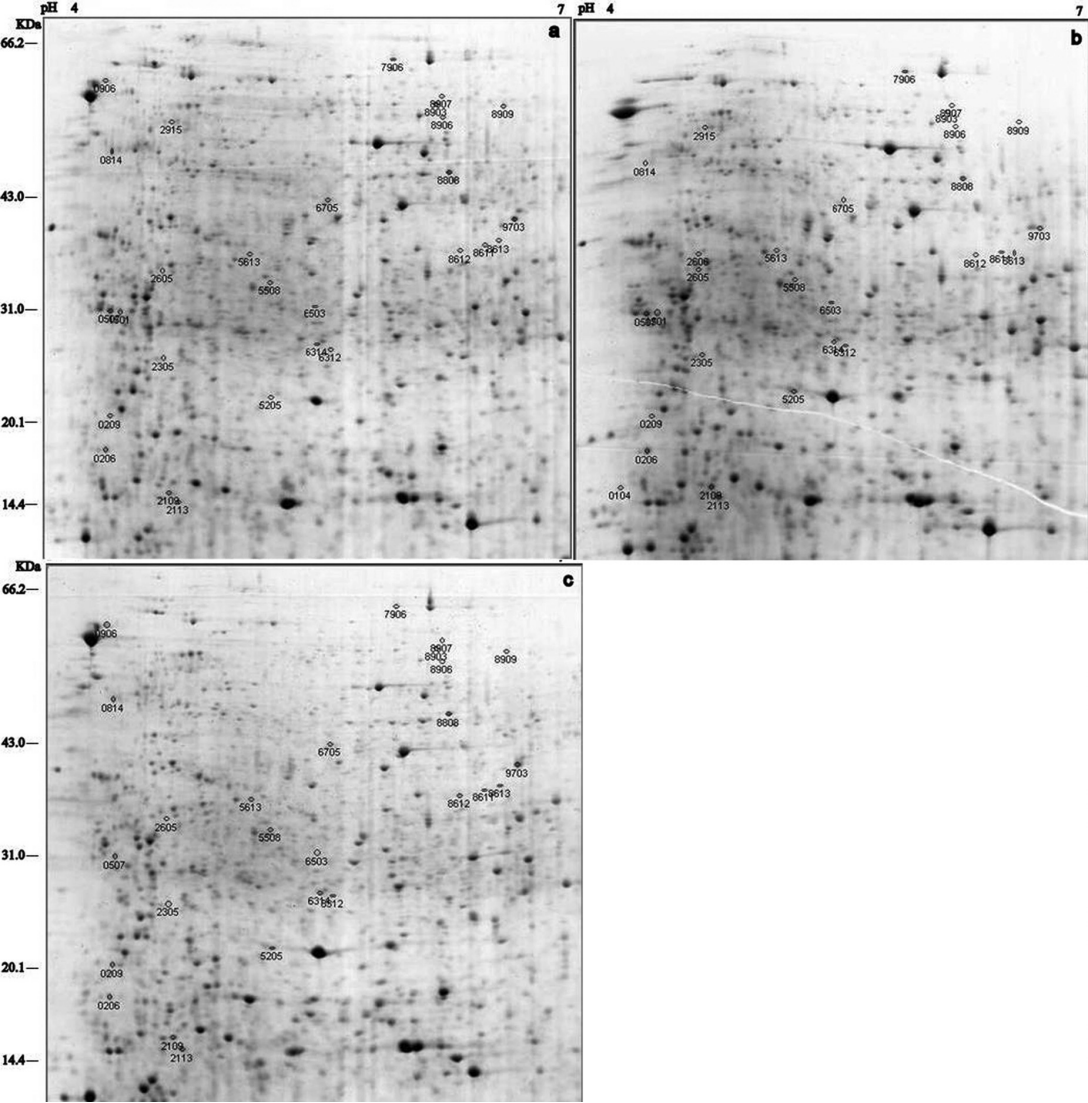
**Two-dimensional electrophoresis gel of separated proteins at three developmental stages**. The pre-albinistic stage (a), albinistic stage (b) and regreening stage (c). Proteins were separated in an IPG strip (pH 4-7) and in the second dimension on a 12.5% gel. Proteins that exhibited a significant expression change (≥ 1.5-fold, ***p***-value ≤ 0.05) from stage a to b while from stage b to c are labeled in the figures.

Two-dimensional electrophoretic analysis of the total protein in leaves at the three developmental stages was performed at least in triplicate and showed a high level of reproducibility. Representative gels are shown in Figure 3. Among all the tested samples, more than 750 protein spots were reproducibly detected with PDQuest 8.0.1 software on CCB G-250-stained gels. Quantitative analysis revealed that 61 protein spots showed a significant (*p *< 0.05) change in intensity by more than 1.5-fold from stage A to B and from stage B to C as well. Three spots (spots 0104, 0906 and 2606) showed qualitative changes. Spots 0104 and 2606 were expressed in the albinistic stage but not in the pre-albinistic and regreening stages. Spot 906 was expressed in the pre-albinistic and regreening stages but not in the albinistic stage. In total, 61 differentially expressed protein spots could be classified into four expressed patterns: pattern I, 31 protein spots showed up-regulation from the pre-albinistic stage to the albinistic stage but down-regulation from the albinistic stage to the regreening stage; pattern II, 21 protein spots showed down-regulation from the pre-albinistic stage to the albinistic stage but up-regulation from the albinistic stage to the regreening stage; pattern III, seven protein spots showed up-regulation from the pre-albinistic stage to the albinistic stage also from the albinistic stage to the regreening stage; and pattern IV, two protein spots showed down-regulation from the pre-albinistic stage to the albinistic stage and also from the albinistic stage to the regreening stage. From the expressed patterns, it seems that the pattern I and II protein spots might be related to the periodic albinism of White leaf No. 1, and pattern III and IV protein spots might be related to the growth of White leaf No. 1.

### Identification of differentially expressed proteins

**Figure 4:**
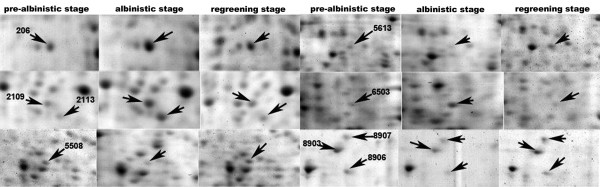
**Enlarged view of the expression patterns of few spots at three developmental stages**.

To understand the albinism mechanism, we combined 2-DE and MALDI-TOF MS/MS to identify the differentially expressed proteins. Thirty differentially expressed protein spots in the expressed patterns I and II were excised from gels, digested in-gel by trypsin and identified by MALDI-TOF/TOF MS. In total, 26 proteins were successfully identified. The results are summarized in Table 1 and the expression patterns of few spots at three developmental stages are enlarged in Figure 4. Five identified spots were annotated either as unnamed and hypothetical proteins or EST in the database. We searched their homologues with BLASTP http://www.ncbi.nlm.nih.gov/BLAST/ using their protein or nucleotide sequences as queries. Five corresponding homologues showing the similarity are shown in Table 2. All shared more than 85% positives with homologues at the amino acid level, implying that they might have similar functions. The remainder of the identified proteins were involved in several functional categories including metabolism of carbon, nitrogen and sulfur, photosynthesis, protein processing, stress defense and RNA processing. Based on putative physiological functions, the identified proteins can be classified into three groups. The first group consists of proteins involved in cell metabolism, including glycolysis, nitrogen and sulfur metabolism, and photosynthesis. The second group consists of regulatory proteins, including proteins involved in protein processing and RNA processing. The third group consists of stress- and detoxification-related proteins, including early light-inducible proteins and isoflavone reductase. The results indicated these physiological processes might play crucial roles in the periodic albinism of White leaf No. 1.

**Table 1:** Differentially expressed proteins identified by MS or MS/MS

**SSP No**.	Protein name	Plant species	Gi number	Mr/pI	Protein score	Pep.^a^	Protein expression^b^
							**A B C**
**Glycolysis and energy**							
206	Phosphoglycerate kinase	*Arabidopsis thaliana*	gi|1022803	23.91/5.05	129	6	
8906	Enolase	*Gossypium barbadense*	gi|33415263	47.87/6.16	97	4	
**Metabolisms of nitrogen and sulfur**							
5508	*S*-adenosylmethionine synthetase	*Camellia sinensis*	gi|75311075	43.23/5.34	324	7	
5613	Glutamine synthetase	*Camellia sinensis*	gi|42733460	39.41/5.52	191	4	
8612	Methylenetetrahydrofolate reductase, 3-partial	*Oryza sativa japonica *group	gi|37718877	42.25/6.10	151	6	
8808	*S*-adenosylmethionine synthetase 3	*Actinidia chinensis*	gi|1709006	39.83/6.2	708	15	
**Metabolism of fatty acid**							
6503	AT2G37660	*Arabidopsis thaliana*	gi|227204455	26.34/5.29	285	6	
**Photosynthesis**							
2113	Ribulose-1,5-bisphosphate carboxylase/oxygenase large subunit	*Spathiostemon javensis*	gi|62003617	52.59/5.96	417	9	
5205	Ribulose-1,5-bisphosphate carboxylase/oxygenase large subunit	*Aruncus dioicus*	gi|533004	52.13/6.04	599	8	
8613	Ribulose 1,5-bisphosphate carboxylase/oxygenase large subunit	*Cylindrocolea recurvifolia*	gi|134274761	48.13/6.08	338	7	
8903	Ribulose-1,5-bisphosphate carboxylase/oxygenase large subunit	*Lycium chinese*	gi|237784017	51.21/6.19	391	16	
8909	Ribulose-1,5-bisphosphate carboxylase/oxygenase large subunit	*Ailanthus integrifolia*	gi|154814186	51.46/6.09	590	17	
**Protein metabolism**							
209	Eukaryotic initiation factor 4A-7	*Nicotiana tabacum*	gi|2500516	40.47/5.17	91	5	
814	Predicted: similar to putative ankyrin-repeat protein	*Vitis vinifera*	gi|255428376	38.06/4.53	404	7	
2915	Lysosomal alpha-mannosidase, putative	*Ricinus communis*	gi|255540059	114.4/5.91	98	2	
5914	Heat shock protein 70	*Cucumis sativus*	gi|6911549	73.59/5.08	73	4	
8907	HSP60-2 (Heat shock protein 60-2); ATP binding	*Arabidopsis thaliana*	gi|30685604	62.34/6.37	93	4	
**RNA metabolism**							
507	29 kDa ribonucleoprotein A, chloroplastic	Nicotiana sylvestris	gi|12230584	29.77/4.75	157	3	
1501	29 kDa ribonucleoprotein B, chloroplastic	*Nicotiana sylvestris*	gi|12230585	31.15/4.92	156	3	
**Stress and defense**							
2109	Early light-induced protein	*Glycine max*	gi|1778823	20.27/9.69	103	3	
9703	Allergenic isoflavone reductase-like protein Bet v 6.0102	*Betula pendula*	gi|10764491	34.17/6.73	205	5	
**Unknown**							
104	EST2066 Tender roots cDNA library of tea plant *Camellia sinensis *cDNA clone	*Camellia sinensis*	gi|212380152	30.31/6.08	78	2	
2605	Unnamed protein product	*Vitis vinifera*	gi|270228367	58.37/5.27	235	4	
2606	Predicted: hypothetical protein	*Vitis vinifera*	gi|225436538	61.93/5.24	149	3	
6314	Predicted: hypothetical protein	*Vitis vinifera*	gi|225462199	26.29/5.58	230	4	
8611	Predicted protein	*Populus trichocarpa*	gi|224092470	48.98/5.40	294	7	

^a ^Number of p matched peptides.

^b ^A: pre-albinistic stage; B: albinitic stage; C: regreening stage.

**Table 2:** Homologues of the unknown proteins

**SSP No**.	NCBI accession No.^a^	Homologue				
		
		NCBI accession No.^b^	Name	Organism	Ident.^c^	Pos.^d^
104	gi|212380152	AAR83862.1	elicitor-inducible protein EIG-J7	*Capsicum annuum*	77%	86%
2605	gi|270228367	BAE71311.1	putative rubisco subunit binding-protein alpha subunit	*Trifolium pratense*	81%	88%
2606	gi|225436538	BAE71311.1	putative rubisco subunit binding-protein alpha subunit	*Trifolium pratense*	86%	93%
6314	gi|225462199	ABL84692.1	glutathione *S*-transferase	*Vitis vinifera*	82%	88%
8611	gi|224092470	NP_568245.1	DEAD/DEAH box helicase, putative	*Arabidopsis thaliana*	95%	98%

^a ^The gi number of the unknown proteins. ^b ^The accession number of the homologues. ^c ^Identities. ^d ^Positives.

### Validation of differentially expressed proteins by western blotting

**Figure 5:**
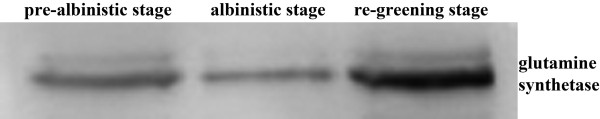
**Western analysis of glutamine synthetase expression level at three developmental stages**.

The proteomic data were preliminary validated by analysis of the protein expression level of glutamine synthetase by western blot, based on the reasonable assumption that equal amounts of total protein from three developmental stages sample were used for western blot analysis[17]. And the results (as shown in Figure 5) suggested that the expressed level of glutamine synthetase varied among three developmental stages. The glutamine synthetase protein levels were decreased at the albinistic stage compared to that at the pre-albinistic stage and then up-regulated at the regreening stage compared to that at the albinistic stage. The western blot results seems to agree well with the proteomic results.

### Gene expression analysis by qPCR

**Figure 6:**
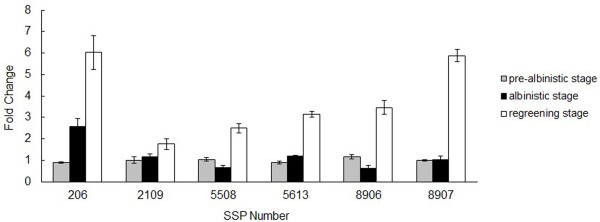
**Real-time PCR analysis of the transcript levels of differentially expressed proteins at three developmental stages**. SSP 206, phosphoglycerate kinase; SSP 2109, Early light-induced protein; SSP 5508, S-adenosylmethionine synthetase; SSP 5613, glutamine synthetase; SSP 8906, enolase; SSP 8907, heat shock protein 60-2.

To investigate the changes in gene expression at the mRNA level, qPCR analysis of six randomly selected identified proteins was performed. The results showed that transcripts of *S*-adenosylmethionine synthetase (SSP 5508) and enolase (SSP 8906) were significantly down-regulated at the albinistic stage to 0.40-fold and 0.45-flod lower than that at the pre-albinistic stage and then significantly up-regulated at the regreening stage to 1.40-fold and 1.97-fold higher than that at the pre-albinistic stage. Phosphoglycerate kinase (SSP 206) and glutamine synthetase (SSP 5613), their transcripts were significantly up-regulated at the albinistic stage to 1.93-fold and 0.33-fold higher than that at the pre-albinistic stage and also significantly up-regulated at the re-greening stage to 5.82-fold and 2.54-fold higher than that at the pre-albinistic stage. The early light-induced protein (SSP 2109) and heat shock protein (HSP) 60-2 (SSP 8907) were not significantly changed between the pre-albinistic stage and the albinistic stage, but they were significantly increased at the re-greening stage to 0.74-fold and 4.86-fold higher than that at the pre-albinistic stage (Figure 6). It was found that the transcription levels of the *S*-adenosylmethionine synthetase and enolase genes were well related to their translation products during the three developmental stages, whilst the levels of the phosphoglycerate kinase, glutamine synthetase and HSP 60-2 transcripts did not correspond with those of their translation products. Thus, overall the mRNA and protein levels were not well correlated.

## Discussion

Albinism has been reported in many higher plant species, such as *Arabidopsis thaliana*, bean and wheat. Transcriptomic analyses have identified many albinism-responsive genes and revealed a complex network involved in the albinism [16,18-20]. A transcriptomic analysis was also used for revealing the regulatory mechanism of changes in leaf color and amino acid levels during leaf development in White leaf No. 1 [14]. However, because of post-transcriptional regulation, the mRNA level does not always correlate well with the protein level [21]. It is insufficient to predict protein expression level from quantitative mRNA data. Therefore, our proteomic analysis of the total proteins provides new insights into the albinism of White leaf No. 1.

In this study, a comparison of the proteome of White leaf No. 1 at three developmental stages was performed. A total of 30 differentially expressed protein spots were excised from gels and processed via MS for identification. Of the 30 selected spots, four spots were not able to be identified from the annotated databases. This is because the complete genome sequence of the tea plant is still unavailable, so the four unidentified protein spots are uncertain.

### Proteins involved in material and energy metabolism

The majority of the identified proteins are involved in primary metabolism and energy metabolism, including metabolism of carbon, nitrogen and sulfur, and photosynthesis. This suggests that such primary and energetic metabolic processes play an important role during the different developmental stages of White leaf No. 1. Two spots were identified as phosphoglycerate kinase (spot 206) and enolase (spot 8906). Their expression changed significantly in the albinistic stage and both are involved in glycolysis. Phosphoglycerate kinase (PGK) is a monomeric enzyme that catalyzes the transfer of the high-energy phosphate group of 1,3-bisphosphoglycerate to ADP, forming ATP and 3-phosphoglycerate. This enzyme is enhanced by cold stress in rice and salt stress in *A. thaliana *[22]. Increase in the expression level of this enzyme at the albinistic stage could indicate an increased production of energy for various cold-stress-related defense processes. Enolase catalyzes the conversion of 2-phosphoglycerate to PEP in glycolysis. Enolase accumulates in a number of plant species in response to a variety of environmental stresses, such as cold and drought [23-25]. However, several reports implicated the protein expression level didn't correlated to enzymatic activity. While the enzymatic activity was increased by stresses, the abundance of enolase protein remained unchanged [26] or markedly decreased [24]. In our experiment, enolase was down-regulated by cold stress, suggesting enolase might be regulated at the post-transcriptional level under cold-stress conditions to regulated the enzymatic activity during the albinism stage. To maintain homeostasis under abnormal growth conditions, the tea plant must activate many resistance mechanisms. Regulation of the abundance of proteins involved in glycolysis might provide additional energy for these processes.

Four protein spots, involved in nitrogen and sulfur metabolism, were identified as *S*-adenosylmethionine synthetase (spots 5508 and 8808), glutamine synthetase (spot 5613) and methylenetetrahydrofolate reductase (spot 8612). *S*-Adenosylmethionine synthetase (SAMS) catalyzes the formation of *S*-adenosylmethionine (SAM) from L-methionine and ATP. SAM is a universal methyl donor in numerous biological reactions and is the methyl donor in the formation of protochlorophyllide from Mg-protoporphyrin. In addition, it is the purine-base methyl donor for biosynthesis of caffeine in tea plants [27]. Decreased expression of SAMS indicated decreased biosynthesis of SAM occurs, perhaps as a consequence of damage to the chloroplasts and reduced photosynthetic carbon assimilation at low temperatures in the albinistic stage. This might be one reason for the low level of caffeine biosynthesis and the change in leaf color in the albinistic stage of White leaf No. 1 [10]. Methylenetetrahydrofolate reductase (MTHFR) is a key enzyme in methionine-folic acid metabolism and serves as a methyl donor for methionine synthesis from homocysteine [28]. The decreased level of MTHFR implied that the biosynthesis of methionine was disturbed with the result that caffeine biosynthesis was suppressed in the albinistic stage of White leaf No. 1. Glutamine synthetase (GS) catalyzes the formation of glutamine from glutamic acid, NH_3 _and ATP. Expression of GS is suppressed by low-temperature stress in a stage albinism line of winter wheat [20] and is induced or reduced by salt stress in leaves of salt-tolerant and salt-sensitive rice, respectively [29]. Because White leaf No. 1 is sensitive to low temperature, GS is down-regulated by low temperature. The enzyme is important in the tea plant to remove toxicity caused by excess ammonium. Through the glutamine synthesis reaction, the tea plant can assimilate a lot of ammonium and transfer the excess to glutamine and other amides. To avoid damage from the high concentration of ammonium under cold stress, the tea plant activates the theanine synthesis pathway and the excess ammonium is stored in theanine. As a result, the theanine concentration is increased in the albinistic stage compared with the regreening stage [10].

Photosynthesis appears to be inhibited in the albinistic stage of White leaf No. 1, as evidenced by suppression of the etioplast-chloroplast transition and damage to grana in the chloroplast. In our proteomic analysis, five protein spots (spots 2113, 5205, 8613, 8903 and 8909) were identified as the RuBisCO large subunit, which displayed heterogeneous expression change patterns during the developmental stages of White leaf No. 1. It should be noted that three spots (spots 2113, 5205 and 8613) might be the products of degradation of the RuBisCO large subunit, because their observed molecular masses were much smaller than their theoretical masses. Similar results were found in rice under cold stress [21], metal stress [30] and ozone stress [31]. The other two spots (spots 8903 and 8909) identified as the RuBisCO large subunit have similar observed and theoretical molecular masses. It seems the two spots represented the intact RuBisCO large subunit and were down-regulated in the albinistic stage of White leaf No. 1. The decline of photosynthetic activity resulted in less production of carbohydrate for glycolysis in leaves at the albinistic stage. Meanwhile, the tea plant need additional energy for activate many resistance mechanisms under abnormal growth conditions. Most of phosphoenolpyruvate (PEP) were shift into tricarboxylic acid cycle (TCA), resulting in less of PEP shift into the shikimic acid pathway and the reduction of polyphenol metabolism in leaves at the albinistic stage of White leaf No.1, but more experimental evidence is needed to confirm this hypothesis.

### Regulatory proteins

Protein and RNA metabolism were affected markedly during the development of White leaf No. 1. Several proteins involved in protein folding (spots 5914 and 8907), synthesis and degradation (spots 209 and 2915), and RNA processing (spots 507 and 1501) changed significantly. Heat-shock proteins/chaperones are responsible for protein folding, assembly, translocation and degradation in cellular processes and contribute to cellular homeostasis under stress conditions [32]. HSP 70 is essential for chloroplast development [33] and is down-regulated in a stage albinism line of winter wheat [20]. In our study, HSP 60 and HSP 70 were down-regulated, which implies that the protein processing system of White leaf No. 1 was disturbed at the albinistic stage. This might be an important reason for the change in leaf color in the albinistic stage. Initiation factor 4A (eIF-4a) plays a crucial role in the eukaryotic translation initiation pathway, and is a ssRNA-dependent ATPase that functions as an RNA helicase. It is also a subunit of the protein complex involved in the initial step of translation initiation, 5'mG cap recognition [34]. The down-regulation of eIF-4A is evidence for disruption of the biosynthesis of new proteins in the albinistic stage of White leaf No. 1. Lysosomal α-mannosidase is a major exoglycosidase in the glycoprotein degradation pathway, involved in the recycling of glycoprotein. In some eukaryotes, a number of unfolded or misfolded glycoproteins are retained in the endoplasmic reticulum (ER) and transferred into the lysosome [35]. These glycoproteins are degraded in the lysosome by a series of hydrolases and lysosomal α-mannosidase, then the release of free polymannose oligosaccharides are utilized for reglycolation or for synthesis of the oligose donor [36]. The up-regulation of lysosomal α-mannosidase indicated that the glycosylation process was disturbed and folding of many glycoproteins failed, and that the tea plant increased expression of this enzyme to aid reglycolation of these glycoproteins in the albinistic stage of White leaf No. 1.

Chloroplast RNA-binding proteins play an important role in RNA processing, transporting and RNA stability. RNA-binding protein CP29 was induced by cold stress in a previous proteomics study of *Arabidopsis *and *Thellungiella *rosette leaves [37,38]. A RNA-binding protein CP-RBP29 was also up-regulated in the albinistic stage of White leaf No. 1. The increase in CP-RBP29 might be a result of enhancing or maintaining chloroplast RNA synthesis, especially for expression of defense-related mRNAs under abnormal conditions [38,39].

### Stress- and detoxification-related proteins

Early light-inducible protein (ELIP), a member of the chlorophyll *a*/*b *binding protein family, has been implicated in the assembly or repair of the photosynthetic machinery during early chloroplast development [40] and is induced by many abiotic stresses [41-43]. The mRNA level of ELIP increases markedly under cold stress in the tea plant [44] and there is a positive correlation between ELIP accumulation and tolerance to chilling-induced photooxidation in barley [45]. In the present investigation, ELIP was up-regulated in the albinistic stage of White leaf No. 1. The damage to chloroplast membrane structure in the albinistic stage of White leaf No. 1 caused by low temperature resulted in reduction of the plant's photosynthetic capacity. The excess light energy could lead to formation of toxic compounds, such as reactive oxygen species. Accumulation of ELIP might protect the tea plant from damage by such toxic compounds. Isoflavone reductase (IFR) (spot 9703) is involved in the production of isoflavone phytoalexins, which utilized phenylalanine as the raw material for the synthetic reaction [46]. In the current study, the down-regulated expression of IFR might be related to the low level of polyphenol metabolism in the albinistic stage of White leaf No. 1 [10]. The low level of polyphenol metabolism lead to less availability of phenylalanine for biosynthesis of isoflavone phytoalexins and the biosynthsis reaction of isoflavone phytoalexins was decreased, so the expression level of IFR was down-regulated at the albinistic stage.

## Conclusions

In summary, proteomics research not only provides new insights into protein expression patterns, but also enables identification of many attractive candidates for further investigation. In this work, the proteome at three developmental stages of the tea cultivar White leaf No. 1 was firstly analyzed. 26 proteins were successfully identified, which related to metabolism of carbon, nitrogen and sulfur, photosynthesis, protein processing, stress defence and RNA processing. In these proteins, only *S*-adenosylmethionine synthetase was identified in previous transcriptomic analysis during the different development stages in White leaf No. 1 [14]. This result suggested that there is a complementary relationship exists between the proteomic analysis and transcriptomic analysis. In addition, the ultrastructural studies revealed that the change in leaf color of White leaf No. 1 might be a consequence of suppression of the etioplast-chloroplast transition and damage to grana in the chloroplast induced by temperature. In general, the results of the ultrastructural analysis and proteomic analysis provide information to improve our understanding of the mechanism of albinism in the tea plant.

## Methods

### Plant material

Leaves of *C. sinensis *cv. White leaf No. 1 were collected from the same 50 tea trees in the tea collection orchard of the Hunan Tea Research Institute, Changsha. Same stage leaves were mixed together and briefly washed with sterile water. The leaves were frozen in liquid nitrogen immediately and stored at -80°C prior to protein extraction.

### Transmission electron microscopic analysis

Leaf samples (about 1 mm^2^) were fixed with 2.5% glutaraldehyde solution overnight at 4°C, then washed with 0.1 M phosphate buffer (pH 7.0) three times. The samples were refixed in 1% (v/v) OsO_4 _solution for 2 h and dehydrated in a graded acetone series. The samples were embedded in Spurr's resin then stained with saturated uranyl acetate in 50% ethanol and 0.2% (w/v) aqueous lead citrate for 15 min each. Sections of 70-90 nm thickness were cut with an EM UC6 microtome (LEICA, Vienna, Austria). The sections were examined and photographed with a JEM-1230 microscope (JEOL, Akishima, Tokyo, Japan).

### Protein extraction

Total protein was extracted according to the method of Jeffries et al. [47] with a slight modification. Leaf samples (3 g) were weighed and ground in liquid nitrogen, then suspended in SDS extraction buffer (0.5% SDS, 10% glycerol, 5% β-mercaptoethanol, 65 mM Tris-HCl [pH 6.8]). After shaking for 1 h, the sample was centrifuged at 12,000 × *g *for 15 min at 4°C. The proteins were precipitated with three to five volumes of acetone containing 10% TCA and 0.7% β-mercaptoethanol at -20°C overnight, then centrifuged at 12,000 × *g *for 15 min at 4°C. The pellet was washed twice with 100% acetone and 80% acetone, respectively, then air-dried for 5 min and resuspended in 20 μL rehydration buffer (7 M urea, 2 M thiourea, 4% CHAPS, 65 mM DTT, 0.2% Bio-Lyte [pH 4-7], and 0.001% bromophenol blue) per 1 mg pellet. Protein content was quantified using the RC DC Protein Assay Kit (Bio-Rad, Hercules, CA, USA).

### Two-dimensional gel electrophoresis

About 1.4 mg protein was dissolved in rehydration buffer and applied to IPG strips (17 cm, pH 4-7, Bio-Rad). The IPG strip was rehydrated for 14 h in 400 μL rehydration buffer containing the protein sample. Isoelectric focusing (IEF) was performed at 20°C using a Protean IEF Cell (Bio-Rad) under the following conditions: 250 V for 1 h with a linear increase in voltage, 500 V for 1 h with a linear increase in voltage, 1000 V for 1 h with a rapid increase in voltage, 10,000 V for 5 h with a linear increase in voltage, and maintained at 10,000 V until a total of 90,000 Volt-hours (Vh) was reached. After IEF, the strips were equilibrated for 15 min in equilibration buffer I (0.375 M Tris-HCl [pH 8.8], 6 M urea, 2% SDS, 20% glycerol, 1% DTT), then re-equilibrated in buffer II containing 2.5% iodoacetamide instead of DTT for 15 min. The strips were transferred onto 12.5% polyacrylamide gels for SDS-PAGE. Electrophoresis was performed using the PROTEAN II xi Cell system (Bio-Rad) at 25 mA per gel for 20 min, followed by 50 mA until the bromophenol blue marker reached the end of the gel. Gels were run in triplicate for each sample. The gels were stained with modified colloidal coomassie brilliant blue (CCB) G-250 [48] and were scanned using Quantity One 4.6.9 (Bio-Rad) with a Bio-Rad GS800 scanner.

### Image analysis and statistical analysis

Image and statistical analysis was performed with PDQuest 8.0.1 (Bio-Rad). After automated detection and spots matching, manual editing was carried out. The gels were normalized by the total density in gel image method. In the quantitative analysis, 1.5 and 0.5 were chosen as the upper and lower limits, respectively. Student's *t*-test and a significance level of 95% were used for the statistical analysis of the gels. Only the spots showing a statistically significant difference in protein abundance between the developmental stages were considered to be differentially expressed spots.

### Protein in-gel digestion and identification by MALDI TOF MS

Protein spots were manually excised from the gel, and washed three times with Milli-Q water and digested as described by Dai et al. [49]. Extracted lyophilized tryptic peptides were dissolved with 0.1% TFA in 50% acetonitrile, then 1 μL of the peptide mixture was mixed with an equal volume of saturated CHCA solution. Mass spectrometric analysis was performed with a MALDI-TOF/TOF mass spectrometer 4800 (Applied Biosystems, Foster City, CA, USA). Data were analyzed using GPS Explorer 3.6 (Applied Biosystems) and MASCOT software (Matrix Science, London, UK). NCBInr and green plants were selected as the database and taxonomy, respectively. The search criteria were mass accuracy of ± 0.3 Da, one missed cleavage site allowed, carbamidomethyl (C) set as a fixed modification, and oxidation of methionine as a variable modification. The identified proteins had to meet three criteria: (1) be among the top hits on the search report; (2) individual ions scores > 44 indicate identity or extensive homology (*p *< 0.05); and (3) more than two peptides matched and a nearly complete y-ion series and complementary b-ion series were present.

### Western blot analysis

Total protein was extracted from leaves at the three developmental stages as above. Fixed amounts of protein were separated by gel electrophoresis, blotted on to PVDF membrane, which was blocked for 1 h at 25°C with 5% wt/vol BSA/TBST (10 mM Tris HCl, pH 7.4, 140 mM NaCl, 0.1% Tween-20) and then incubated with the polyclonal antibody for glutamine synthetase (1:10000, Agrisera, AS08295) at 4°C overnight. After washing with TBST, the membranes were incubated with the appropriate secondary antibodies for 1 h at 37°C and detected by immuno-staining. After the membranes were scanned, the signal intensity of each band was determined using FluorChem FC2(Alpha Innotech Co., Ltd).

### Quantitative real-time PCR analysis

Total RNA for real-time PCR (qPCR) analysis was extracted from leaves at the three developmental stages using the RNeasy Plant Mini Kit (Qiagen, Hilden, Germany) and Free DNase Set (Qiagen). cDNA was synthesized from the total RNA (1 μg) using the oligo(dT)18 primer and Moloney Murine Leukemia Virus Reverse Transcriptase (Promega, Madison, WI, USA) according to the manufacturer's instructions. The primers (Table 3) for qPCR were designed using Beacon Designer 7.0 software according to the cDNA sequences. Reactions were carried out with the Rotor-Gene Q 6200 Real-Time PCR System (Qiagen) using three-step cycling conditions of 95°C for 10 min, followed by 45 cycles of 95°C for 10 s, 52-55°C for 15-25 s and 72°C for 20 s. The reaction mixture (20 μL) contained 1 μL cDNA solution, 10 μL Platinum SYBR^® ^Green qPCR SuperMix-UDG (Invitrogen, Carlsbad, CA, USA) and 6 μM of each primer. The reactions were performed in triplicate, and the results were averaged. *GAPDH *was used as the reference gene. The relative gene expression was evaluated using the comparative cycle threshold method [50]. Statistical analysis was performed using ANOVA and Post Hoc test with a *p *value < 0.05 being accepted as significant.

**Table 3:** Primer sequences used in qPCR

SSP number	Forward primer (5'-3')	Reverse primer (5 '-3 ')
206	TCTGCTTGGTGGTGGAAT	CATCAGGAGCGAACTTGTC
5508	TGATGAGATTGCTGCTGAT	GTTGAGGTGGAAGATGGT
5613	ATGCTGCCAAGATATTCA	AAGTGTACTCCTGCTCTA
8906	GTTGTTATTGGAATGGATGT	GGCTACGAATGACTTGTA
8907	GGTGGTGGTGTTGCTCTTTT	GTTCCAAAAGCTTGCCTACG
GAPDH	TTGGCATCGTTGAGGGTCT	CAGTGGGAACACGGAAAGC

## Competing interests

The authors declare that they have no competing interests.

## Authors' contributions

QL carried out the 2-DE experiments and mass spectrometry analysis. JH participated in the optimization of 2-DE protocol. SL participated in the design of the study. JL participated in the qPCR experiments. XY participated in the western analysis. YL participated in the protein identification. ZL conceived of the study, and participated in its design and coordination. All authors read and approved the final manuscript.
